# Ecological Stoichiometric Characteristics of Plant–Litter–Soil Among Different Forest Stands in a Limestone Region of China

**DOI:** 10.3390/plants14121758

**Published:** 2025-06-08

**Authors:** Yeqiao Wang, Haochuan Tu, Jingjing Zheng, Xiongjie Li, Guibin Wang, Jing Guo

**Affiliations:** State Key Laboratory of Tree Genetics and Breeding, Co-Innovation Center for Sustainable Forestry in Southern China, Nanjing Forestry University, Nanjing 210037, China; wyq8513@163.com (Y.W.); thch0615@163.com (H.T.); jjing@njfu.edu.cn (J.Z.); lixiongjie@njfu.edu.cn (X.L.); guibinwang99@163.com (G.W.)

**Keywords:** ecological stoichiometry, limestone region, nutrient limitation, plant–litter–soil continuum

## Abstract

The transformation of degraded stands represents an essential strategy for enhancing stand productivity and optimizing site adaptability. This study examined four typical monoculture forest stands transformed from underperforming *Platycladus orientalis* (*PO*) forests in the limestone area of Xuzhou, China: *Acer pictum subsp. mono* (*AP*), *Pistacia chinensis* (*PC*), *Ligustrum lucidum* (*LL*), and *Firmiana simplex* (*FS*). The contents of carbon (C), nitrogen (N), and phosphorus (P), along with the C:N:P stoichiometric ratios, were analyzed in plants (leaves and fine roots), litter, and soil. The relationships among these components and their main influencing factors were explored. The results indicated that *FS* leaves contained higher levels of N and P, whereas *LL* litter presented significantly elevated C:N and N:P ratios in comparison with those of the other forest stands (*p* < 0.05). With the exception of *FS*, leaves displayed lower P than fine roots, which presented pronounced P enrichment. The soil C, N, and P contents decreased with depth, with both the forest stand and depth significantly impacting the soil stoichiometry (*p* < 0.01). Redundancy analysis identified available potassium, total nitrogen, and microbial biomass carbon in the soil as key factors influencing the stoichiometric characteristics of the leaf–fine root–litter continuum. Collectively, the leaf N:P ratios (>16) and low soil P contents indicate that plantation growth was primarily constrained by P limitation. In response, *AP*, *PC*, and *LL* allocate more P to fine roots to adapt to the environment.

## 1. Introduction

Afforestation serves as an effective strategy to increase carbon (C) sequestration, mitigate global warming, and restore ecosystems. Although the global forest area continues to decrease, the rate of loss has slowed in recent years, attributable to efforts in forest restoration across regions. Australia and India recorded annual net increases in forest area of 446,000 and 266,000 hectares, accounting for 0.34% and 0.38% of the global net increase, respectively (FAO. 2024. The State of the World’s Forests 2024—Forest-sector innovations towards a more sustainable future. Rome, FAO. https://doi.org/10.4060/cd1211en, accessed on 28 May 2025). In recent decades, China’s large-scale afforestation initiatives have also achieved remarkable accomplishments. Chinese forest coverage experienced a net increase of 4.75%, with annual forest restoration rates averaging 4.07 million hectares per year from 2000 to 2015 [1]. Although this rate slowed to 2.26 million hectares per year from 2015 to 2022, the forest area is expected to continue expanding [1]. However, the extensive long-term expansion of plantations, coupled with the rigid constraints of China’s 120-million-hectare cultivated land preservation policy and forest-to-farmland reconversion initiatives, has precipitated a continuous shrinkage in high-quality land resources for afforestation. Additionally, challenges such as inappropriate species or site selection, irrational stand structure, and insufficient scientific management have led to widespread issues in plantations, including reduced productivity, limited ecological functions, stand degradation, and mortality, resulting in extensive areas of low-quality forests [2].

Consequently, current plantation management strategies urgently need to shift from area expansion to quality improvement. Forest management is a crucial means to maintain the long-term health and productivity of forests, playing a vital role in ecological balance, biodiversity conservation, climate change, and sustainable resource utilization. A critical challenge lies in optimizing the ecological benefits of existing stands [3]. It is important in difficult sites with harsh, unbalanced, and poorly regulated environmental conditions where early afforestation faces problems such as unreasonable density, unsuitable species selection, and improper management (lack of thinning, dead wood removal, pruning, etc.) [2,4,5]. Moreover, the blind pursuit of afforestation areas has neglected the sustainable development of forest stands. Therefore, the transformation of existing stands on difficult sites is an urgent task.

Ecological stoichiometry integrates ecology and stoichiometry to examine energy and elemental balance across biological systems, spanning from molecules to ecosystems [6,7]. It has been widely applied in various research fields, such as climate change, soil microbial enzymes, litter decomposition, and leaf economics [8,9,10,11]. Its application in forest ecosystems has also been a popular research area, and stoichiometric ratios serve as useful indicators for plant growth, soil nutrient availability, and litter decomposition rates [12]. The plant growth rate is negatively correlated with the leaf C: phosphorus (P) and nitrogen (N):P ratios [13,14], and stoichiometric threshold ratios can determine nutrient limitations in plants, soils, and microbes [15,16], providing a new perspective on plant–soil interactions [17]. The initial C:N ratio of litter largely determines its decomposition rate, which in turn affects nutrient return rates to plants and the soil C sequestration capacity [18]. Differences in C allocation strategies among organs and nutrient requirements across forest stands influence the ecological stoichiometric characteristics of ecosystems, ultimately affecting biochemical processes and ecosystem functions [19]. Hence, ecological stoichiometric characteristics are used to analyze aboveground–belowground linkages and assess nutrient use efficiency and nutrient limitations [20].

Plants and soil maintain relatively stable and balanced C:N:P stoichiometry via nutrient uptake and litter decomposition, forming a continuous “plant–litter–soil” system [21]. Previous studies have mostly focused on individual components, such as roots, stems, leaves, soil, extracellular enzymes, and litter [22,23,24]. However, the lack of integrated exploration of multiple components has limited the understanding of C, N, and P balance and cycling in forest ecosystems. The “plant–litter–soil” continuum is a complex, tightly interconnected organic system that jointly participates in the functions and processes of forests [25], and there are varying degrees of correlation in terms of their ecological stoichiometry. For example, studies on typical plantations on the Loess Plateau revealed strong N coupling among leaves, roots, litter, and soil, whereas P exhibited stronger coupling between leaves, roots, and litter [26]. Comprehensive investigations of the stoichiometric characteristics of the “plant–litter–soil” continuum will enhance our multidimensional understanding of forest ecosystem functional dynamics and underlying mechanisms [27,28]. Recent studies have largely concentrated on northern arid regions or southern forests, with less attention given to artificial plantations in limestone areas [26,29,30,31]. Research on forest continuum stoichiometry in degraded habitats can better elucidate elemental cycling patterns under nutrient-poor conditions, reflecting species growth adaptability and interactions with soil.

Limestone hills represent one of the typical difficult sites in Jiangsu Province, characterized by shallow soils and severe soil erosion. Since the 1960s, afforestation programs supported by Chinese greening projects have been implemented, with *Platycladus orientalis* (*PO*) being the main plantation species. However, owing to excessive planting density, simplified stand structure, and continuous mortality of some plants, large areas of low-quality and inefficient forests have formed. Over a decade ago, a forest stand improvement project was initiated locally, where clearcutting and the introduction of multiple tree species were implemented. This has led to the establishment of large areas of artificial forests with diverse species compositions. While numerous studies have documented the ecological stoichiometry of plant organs, litter, and soil along with environmental drivers in analogous karst rocky regions [32,33,34], the ecological effectiveness of forest stand improvement in limestone hills remains unexplored. Therefore, this study analyzes the ecological functions of forest stands from the perspective of ecological stoichiometry to provide novel insights for forest stand improvement. Focusing on typical plantations in Xuzhou’s limestone hills in China, this research includes (1) the stoichiometric characteristics of plants (leaves and fine roots), litter, and soil (0–5 cm, 5–10 cm, and 10–20 cm) across different forest stand types; (2) the C:N:P correlations among plants, litter, and soil in plantations; and (3) the linkages and key influencing factors within the plant–litter–soil continuum. By systematically analyzing stoichiometric patterns in various forest stands, this study aims to reveal the elemental coupling mechanisms within the plant–litter–soil continuum under degraded habitats and the effects of forest stand improvement on the stoichiometric balance of ecosystems.

## 2. Results

### 2.1. Ecological Stoichiometry in Plants

**Figure 1:**
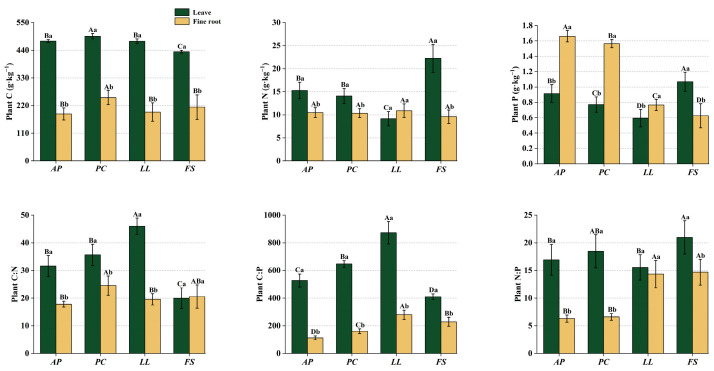
Stoichiometric characteristics of leaves and fine roots across forest stands. Different uppercase letters indicate significant differences among forest stands for the same organ (*p* < 0.05); different lowercase letters denote significant differences between organs within the same forest stand (*p* < 0.05); *Acer pictum subsp. mono* (*AP*), *Pistacia chinensis* (*PC*), *Ligustrum lucidum* (*LL*), and *Firmiana simplex* (*FS*).

The C contents of leaves and fine roots across forest stands ranged from 434.33 to 496.08 g·kg^−1^ and 186.62 to 251.91 g·kg^−1^, respectively, with N contents ranging from 9.14 to 22.25 g·kg^−1^ (leaves) and 9.58 to 10.88 g·kg^−1^ (roots), and P contents ranging from 0.59 to 1.07 g·kg^−1^ (leaves) and 0.66 to 1.66 g·kg^−1^ (roots) (Figure 1). The C:N ratios of leaves and fine roots were 19.85–53.42 and 17.82–24.54, the N:P ratios were 15.55–20.98 and 6.30–14.70, and the C:P ratios were 411.19–822.22 and 112.33–300.35, respectively. 

*Pistacia chinensis* (*PC*) had significantly greater C content in leaves and fine roots than the other forest stands (*p* < 0.05), whereas *Firmiana simplex* (*FS*) presented the lowest leaf C content (*p* < 0.05). *FS* presented the highest leaf N content among all the forest stands (*p* < 0.05), whereas *Ligustrum lucidum* (*LL*) presented the lowest leaf N content (*p* < 0.05). *FS* presented the highest P content in the leaves but the lowest P content in the fine roots.

The leaf C:N ratios varied significantly among the forest stands, with *LL* exhibiting the highest values and *FS* the lowest (*p* < 0.05). Compared with the *Acer pictum subsp. mono* (*AP*) and *LL* stands, the *PC* stands presented elevated fine root C:N ratios, whereas *FS* presented higher leaf N:P ratios than the other stands (*p* < 0.05). *AP* and *PC* presented lower fine root N:P ratios than *LL* and *FS* (*p* < 0.05). Significant differences in the leaf and fine root C:P ratios were observed across all stands (*p* < 0.05). Overall, the C:N:P ratios in leaves and fine roots differed markedly between stand types. Compared with fine roots, leaves consistently contained more C and N (*p* < 0.05), whereas fine roots presented significant P enrichment.

### 2.2. Ecological Stoichiometry in Litter

**Figure 2:**
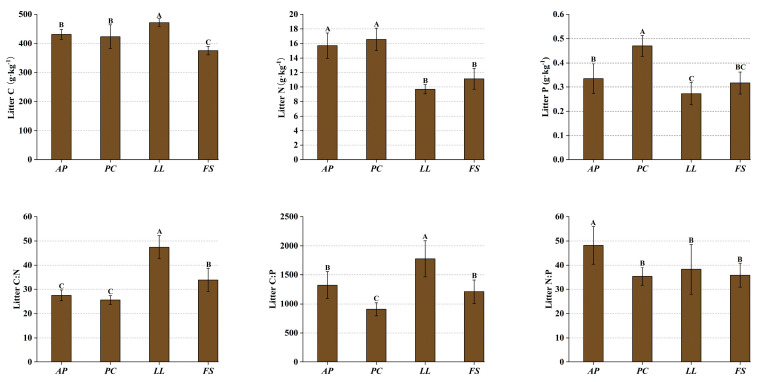
Stoichiometric characteristics of litter across forest stands. Different letters indicate significant differences between forest stands (*p* < 0.05).

Litter C:N:P stoichiometry differed significantly among the forest stands (*p* < 0.05) (Figure 2). The C content of the litter ranged from 375.18 to 471.85 g·kg^−1^, whereas the N and P contents ranged from 9.70 to 16.56 g·kg^−1^ and 0.27 to 0.47 g·kg^−1^, respectively. The C:N ratio ranged from 25.62 to 48.80, the C:P ratio ranged from 907.26 to 1776.59, and the N:P ratio ranged from 35.42 to 48.19. *LL* presented the highest litter C:N and N:P ratios, which were significantly greater than those of other forest stands (*p* < 0.05). *AP* and *FS* showed no significant difference in litter C:P ratio (*p* > 0.05), but both were significantly lower than that of *LL* (*p* < 0.05).

### 2.3. Ecological Stoichiometry in Soil

**Figure 3:**
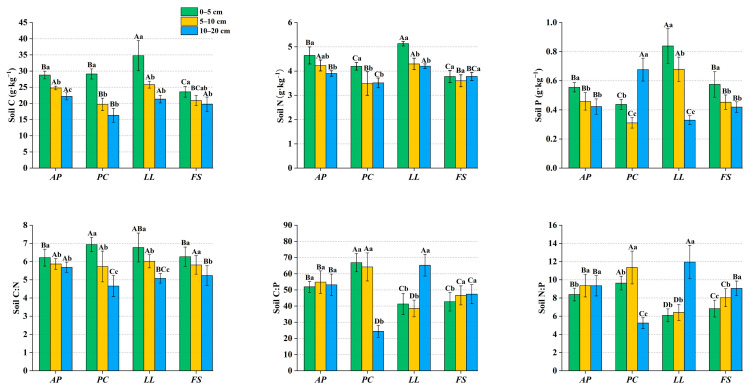
Stoichiometric characteristics of soil across forest stands. Different uppercase letters indicate significant differences between forest stands within the same soil layer (*p* < 0.05), whereas different lowercase letters indicate significant differences within the same forest stand between different soil layers (*p* < 0.05).

Forest stand and soil depth had highly significant effects on the soil C, N, and P contents and stoichiometric ratios, and their interaction was highly significant (*p* < 0.05) (Table 1). The soil C, N, and P contents generally decreased with depth, indicating pronounced “nutrient surface accumulation” (Figure 3). Notably, the soil P content in *PC* exhibited an anomalous increase in deeper layers (10–20 cm). Across all the soil depths, the stoichiometric characteristics differed significantly among the forest stands (*p* < 0.05). The soil C content in the study area ranged from 16.33 to 34.75 g·kg^−1^, the N content ranged from 3.49 to 5.13 g·kg^−1^, and the P content ranged from 0.31 to 0.85 g·kg^−1^. In the 0–10 cm layer, *LL* showed significantly higher C, N, and P contents than the other forest stands (*p* < 0.05). In the 10–20 cm layer, *PC* had significantly lower C and P contents, whereas *LL* maintained a significantly higher N content than the other forest stands (*p* < 0.05). The soil C:N ratios ranged from 4.65 to 6.93 and decreased with depth. In the 0–5 cm layer, *PC* presented a significantly greater C:N ratio than *AP* and *FS* (*p* < 0.05), whereas this pattern was reversed in the 10–20 cm layer. The N:P ratios varied between 5.23 and 12.85, with *LL* presenting the lowest N:P ratio in the 0–10 cm layer but the highest N:P ratio in the 10–20 cm layer, significantly exceeding those of the other forest stands (*p* < 0.05). The C:P ratios ranged from 24.19 to 66.90, with *PC* showing significantly higher C:P ratios than the other forest stands in the 0–5 cm layer (*p* < 0.05). Significant differences in C:P ratios among the forest stands persisted throughout the 5–20 cm soil layers (*p* < 0.05).

### 2.4. Relationship Among Plants, Litter, and Soil in Artificial Forests

#### 2.4.1. Analysis of Stoichiometry Between Plants, Litter, and Soil

**Figure 4:**
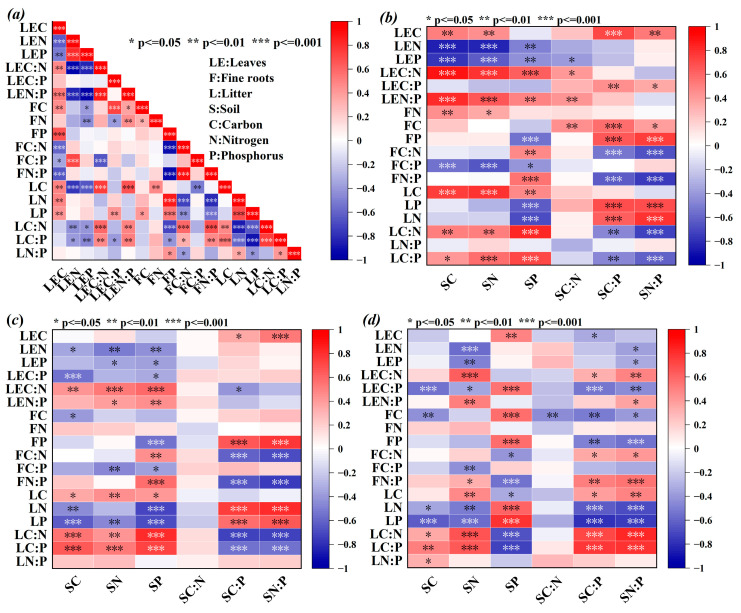
(**a**) Leaf–fine root–litter stoichiometry relationship; (**b**) leaf, fine root, litter, and 0–5 cm soil stoichiometric relationships; (**c**) leaf, fine root, litter, and 5–10 cm stoichiometric relationships; (**d**) leaf, fine root, litter, and 10–20 cm stoichiometric relationships. LE, leaves; F, fine roots; L, litter; S, soil; C, carbon; N, nitrogen; P, phosphorus; symbol: *, *p* ≤ 0.05; **, *p* ≤ 0.01; ***, *p* ≤ 0.001. The closer a cell’s color gets to the red at the top of the scale, the stronger the positive correlation; the closer it gets to the blue at the bottom, the stronger the negative correlation.

Among the aboveground components, leaf C was positively correlated with fine root C, litter C, and litter P (*p* < 0.01) but significantly negatively correlated with fine root C:N:P (*p* < 0.05, Figure 4a). Leaf N was positively correlated with fine root C:P (*p* < 0.01) and negatively correlated with litter C:N and C:P ratios (*p* < 0.05). The leaf C:N ratio was linked to fine root and litter C:P ratios, whereas the leaf N:P was strongly positively correlated with the fine root C and N and litter C:N (*p* < 0.05). Fine root C was positively correlated with litter P (*p* < 0.05), and fine root N was positively correlated with litter C (*p* < 0.01). Fine root P was positively correlated with litter N and P (*p* < 0.01) but negatively correlated with litter C:N and C:P ratios (*p* < 0.05).

In terms of the correlations between the aboveground and belowground components (Figure 4b–d), the leaf C content was significantly positively correlated with the soil C:P ratio and N:P ratio (*p* < 0.05). Leaf N was negatively correlated with 0–10 cm soil C, N, and P and 10–20 cm soil N (*p* < 0.05). Leaf P and C:N were negatively related to soil N, whereas leaf N:P was positively correlated with soil N (*p* < 0.05). Fine root P showed contrasting relationships: strong negative correlations with 0–10 cm soil P but positive correlations with soil C:P and N:P (*p* < 0.01). Conversely, fine root C:N and N:P ratios were positively correlated with 0–10 cm soil P and negatively correlated with soil C:P and N:P ratios (*p* < 0.01). In the 0–10 cm soil layer, litter N and P were negatively correlated with soil P (*p* < 0.01) and positively correlated with soil C:P and N:P (*p* < 0.05). Litter C:N and C:P ratios were positively correlated with soil P but negatively correlated with soil C:P and N:P ratios (*p* < 0.01) in the 0–10 cm layer. In contrast, these relationships reversed in the 10–20 cm layer (*p* < 0.01).

#### 2.4.2. RDA of Aboveground Stoichiometry and Soil Properties

**Figure 5:**
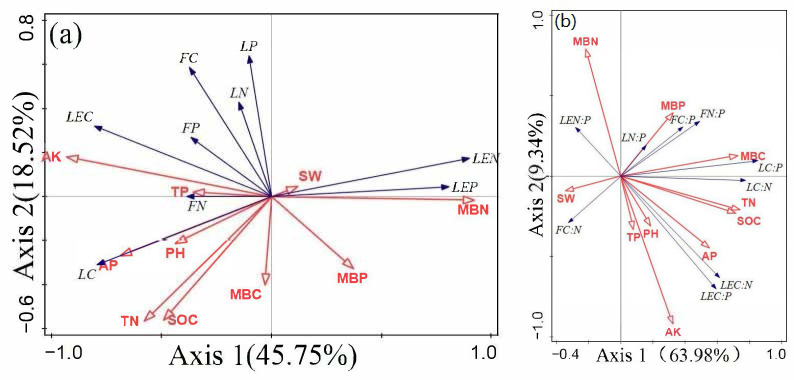
(**a**) RDA of aboveground C, N, and P with respect to soil properties. (**b**) RDA of aboveground C:N:P ratios with respect to soil properties. Blue and red arrows represent the response variables and explanatory variables, respectively. LE, leaves; F, fine roots; L, litter; SW, soil water content; SOC, soil organic carbon; TN, total nitrogen; TP, total phosphorus; AP, available phosphorus; AK, available potassium; MBC, microbial biomass carbon; MBN, microbial biomass nitrogen; MBP, microbial biomass phosphorus.

RDA was conducted with the aboveground components (leaves, fine roots, and litter), the C, N, and P contents, and stoichiometric ratios as response variables and the soil properties as explanatory variables. The results revealed that the first and second ordination axes explained 45.75% and 18.52% of the variation in aboveground C, N, and P, respectively (Figure 5a). Among the soil properties, AK (*p* = 0.002), TN (*p* = 0.002), MBN (*p* = 0.026), and MBC (*p* = 0.034) explained 40.3%, 11.6%, 4.8%, and 4.5% of the variation in the aboveground C, N, and P contents, respectively. These soil properties were identified as key factors influencing the variation in aboveground C, N, and P contents in forests.

As shown in Figure 5b, the first and second ordination axes explained 63.98% and 9.34% of the variation in the aboveground C:N:P ratios, respectively. TN (*p* = 0.002), MBC (*p* = 0.002), AK (*p* = 0.01), and MBP (*p* = 0.01) were identified as key factors influencing the variation in the aboveground C:N:P ratios in forests. These factors explained 35.8%, 18.2%, 8.3%, and 6% of the variation in the response variables, respectively.

## 3. Discussion

### 3.1. Plant Stoichiometry of Different Forest Stands

The appropriate nutrient concentration and stoichiometric characteristics of plants constitute a fundamental prerequisite for optimizing their photosynthetic performance and biomass allocation. This dynamic equilibrium critically underpins the resilience of ecosystem multifunctionality by mediating energy transduction efficiency among ecosystem components [6]. In this study, all forest stands except *FS* presented higher leaf C contents than the Chinese community average (436.80 g·kg^−1^) and global forest baseline (455.10 g·kg^−1^) [35,36]. This elevation likely reflects adaptive structural C allocation (e.g., lignin and cellulose) to enhance stress resistance in limestone environments. For example, plant biomass accounts for 80% of the total C pool in Xishuangbanna limestone forest ecosystems, suggesting preferential C allocation to structural storage that indirectly elevates the leaf C content [37]. Therefore, this mechanism may indirectly contribute to the increased leaf C content. Except for *FS*, the leaf N content in the other forest stands was lower than the Chinese average for plant communities (20.24 g·kg^−1^) and global levels (20.09 g·kg^−1^) [38,39] and lower than the forest levels in arid karst regions (16.70 g·kg^−1^) [40,41]. *FS*, with the highest leaf P content, was similar to the average level of forests in the karst region of China, but the other forest stands had lower leaf P contents [42,43]. Compared with rock desertification areas and evergreen broadleaf forests, *LL* areas presented the lowest leaf P content (0.69 g·kg^−1^, 0.71 g·kg^−1^) [41,43]. This can be analyzed from two perspectives: leaf N and P contents are closely related to soil N and P contents [44], and in limestone hills, a limited soil supply results in lower leaf N and P levels in plants; on the other hand, from a plant functional trait perspective, plants growing in nutrient-poor habitats actively reduce their investment in leaf N and P to ensure survival [45]. On the basis of the above findings and considering that *FS* leaves have significantly higher N and P contents than the other forest stands (Figure 1), it can be inferred that *FS* focuses more on the allocation of N and P to leaves, possibly at the expense of structural C storage. This is because *FS* is a fast-growing species that requires more nutrients for growth and photosynthesis, with higher N and P demands [46]. Additionally, more nutrients may need to be transported from fine roots to leaves [47]. In this study, the fine root C content across all stands was lower than the global average (440 g·kg^−1^), whereas the N content fell within the typical range (9.9–11.2 g·kg^−1^); *LL* and *FS* presented fine root P levels within the normal range (0.55–0.85 g·kg^−1^), whereas *AP* and *PC* presented significantly higher P content [48]. A comparison of the fine root and leaf N and P contents (Figure 1) revealed that *AP*, *PC*, and *LL* fine roots presented significant P enrichment and stored more P than the leaves. Typically, leaves are more metabolically active and have a stronger capacity for nutrient absorption, which is why leaf element contents are usually higher. This anomalous phenomenon reflects the characteristics of different tree species. In addition to fast-growing *FS*, other species adopt a more conservative survival strategy, tending to reduce investment in leaves and store P in roots [45,49,50,51]. Given that only *LL* presented a greater fine root N content than the leaves, its conservative strategy may be more pronounced. Additionally, the lower C content in fine roots suggests that they may have undergone more metabolic activities. This is due to the shorter lifespan and faster turnover rate of fine roots [52], which require more C to support a variety of physiological metabolic activities.

Elemental stoichiometric ratios, which serve as stabilized indicators of environmental adaptation, effectively reflect plant capacities for C utilization and nutrient acquisition [53,54]. Consistent with the growth rate hypothesis [14], *FS* presented significantly lower leaf C:N and C:P ratios than the other stands (Figure 1), indicating faster growth rates that align with its species traits. Higher C:nutrient ratios typically indicate enhanced C assimilation and nutrient use efficiency [13,55]. The C:N and C:P ratios of fine roots in all forest stands were lower than the geometric mean values of fine roots in Chinese plant species [56], suggesting that the plantations exhibit relatively low nutrient absorption efficiency. Among the four stands, *LL* demonstrated relatively greater leaf and fine root nutrient efficiency. Additionally, per the N:P threshold theory, foliar N:P ratios exceeding 16 across all forest stands, paired with average N content in leaves and fine roots [15,57], suggest that these stands are likely P-limited.

### 3.2. Litter Stoichiometry of Different Forest Stands

In forest ecosystems, more than 80% of photosynthetically fixed C enters soil via litter, whereas the N and P absorbed by plants are derived primarily from litter decomposition—a pivotal process that governs soil nutrient replenishment and sustains organic matter accumulation and nutrient cycling [58,59]. Nutrient resorption constitutes a critical adaptation for plants to conserve nutrients, especially in nutrient-poor environments [60]. In the method proposed by Killingbeck [61], maximal resorption occurs when senesced-leaf N and P concentrations fall below 7 g·kg^−1^ and 0.5 g·kg^−1^, respectively, whereas values exceeding 10 g·kg^−1^ (N) and 0.8 g·kg^−1^ (P) indicate low resorption efficiency. Our findings demonstrate universal attainment of maximal P resorption across stands, in contrast to suboptimal N resorption, which is highest in *LL* (Figure 1). Comparative analysis with Loess Plateau plantations revealed comparable litter N levels but significantly lower P concentrations [62]. This suggests that in all the forest stands, the leaf N resorption efficiency is lower than the P resorption efficiency, indicating that plants might have a greater capacity for conserving P.

The litter C:N ratio is significantly negatively correlated with the decomposition rate, where a lower C:N ratio typically accelerates breakdown [18,63]. Compared with the other stands, the *LL* stands presented higher C:N and C:P ratios (Figure 2), indicating relatively slower decomposition rates. This may be due to the leathery leaves of *LL*, which are harder to break down naturally and thus more resistant to decomposition by decomposers. In contrast, the leaves of *AP* and *PC*, which have lower C:N ratios, are smaller and more paper-like, making them easier to leach and fragment, leading to faster decomposition rates. Additionally, studies have indicated that plants with high nutrient resorption efficiency produce litter with relatively high C:P ratios, imposing microbial P limitations that further suppress decomposition activity [64]. However, the slower decomposition of *LL* litter is unfavorable for organic matter accumulation in forest soils and nutrient return [65]. Therefore, large-scale *LL* afforestation may negatively impact soil C sequestration and nutrient supply capabilities.

### 3.3. Soil Stoichiometry of Different Forest Stands

The 0–20 cm soil layers across the four forest stands exhibited markedly higher N contents (exceeding China’s average of 1.88 g·kg^−1^) but lower P levels except in *LL* (<0.78 g·kg^−1^) [66]. Notably, the 0–5 cm soil C content in *AP*, *PC*, and *LL* is higher than the Chinese average (24.56 g·kg^−1^) [66]. Compared with forest ecosystems in other regions of China [31,67,68], the soil in this study overall showed higher C and N contents but lower P contents. As a relatively infertile site, this phenomenon may seem unusual but is likely related to the soil organic matter decomposition process. Soil C:N ratios greater than 25 are considered indicative of organic matter accumulation being greater than decomposition [66]. Our results revealed that the C:N ratio in the soil across different forest stands and soil layers was less than 8 (Figure 3), suggesting that organic matter decomposition was more complete. A more important factor could be the region’s long history of afforestation. Several studies have shown that long-term afforestation can significantly increase SOC and TN [29,69,70]. The P limitation in this region results from multiple factors. First, the primary source of P in soil is the parent material, and the limestone itself has a relatively low P content, which leads to a lower P content in the soils in which it has developed [71]. Second, limestone soils are rich in calcium carbonate, and the pH is usually relatively high, making phosphorus prone to binding with calcium and magnesium ions, forming insoluble calcium phosphate precipitates that are difficult to absorb and utilize [72]. Finally, the shallow soil layers and high proportion of bare rock further limit the accumulation of P due to leaching effects [73].

The forest stand type significantly influenced the soil C:N:P stoichiometry (*p* < 0.05), with substantial variation in the C:N:P ratios across different forest stands. This variation could be due to differences in how different plant species absorb elements from the soil and release them through litter decomposition, thereby altering the C:N:P ratio [74]. Although the soil layers are shallow, the soil C, N, and P contents gradually decrease with increasing soil depth (Figure 3), which is consistent with the findings of previous studies [25,31,67]. This pattern also aligns with the observation that deep soil accumulates less C and N than surface soils and requires more time for accumulation [75]. A study by Yang and Liu [76] revealed that the C:N ratio decreases with soil depth, whereas the C:P and N:P ratios increase gradually. The C:N and N:P ratios observed in this study across different soil layers followed this trend. Species identity and soil depth critically maintain C:N:P stoichiometry through litter inputs [77]. The soil C:N ratio serves as an indicator of N mineralization capacity, with lower ratios correlating to faster organic matter decomposition [31]. All the soil layers presented C:N ratios below the global average (14.3) [78], suggesting accelerated N mineralization and decomposition processes. This likely contributed to the elevated soil C and N levels observed locally. Notably, regional soil N:P ratios exceed China’s terrestrial average (5.2) [66], indirectly reflecting the high soil N content and low P content.

### 3.4. Analysis of Stoichiometric Relationships Among Plants, Litter, and Soil

C, N, and P in the continuum are tightly coupled [62,79]. Plant N and P availability is constrained by the soil supply, where shifts in soil nutrient levels directly regulate plant uptake efficiency, thereby altering growth and metabolic processes [80]. Root stoichiometry is governed by soil nutrient availability [81]. In the study area, low nutrient levels constrain N and P uptake, diminishing nutrient cycling and metabolic activity and thereby impairing plant–soil feedback mechanisms [82]. In the 0–10 cm soil layer (Figure 4b,c), leaf N was negatively correlated with soil N, while root N was positively correlated with soil N (*p* < 0.05). These relationships suggest that plants primarily source soil-derived N, allocating it preferentially to leaves to optimize growth. Fine root P was negatively correlated with soil P but positively correlated with soil C:P and N:P (*p* < 0.05); however, these relationships were reversed in the 10–20 cm soil layer. Plants preferentially absorb N and P from surface soils via fine roots, which aligns with vertical nutrient declines. Moreover, litter P was positively related to fine root P but negatively related to root C:N and N:P ratios, indicating that plants primarily acquire P through fine root uptake from soil sources rather than from litter, with most litter P retained during decomposition processes [83]. Similar to fine roots, litter and soil C:N:P showed contrasting correlations across different soil layers, suggesting that litter decomposition rates regulate C input and nutrient return to indirectly drive organic C, N, and P accumulation in surface soils, while deeper layers may remain unaffected.

The RDA results revealed that AK, TN, and MBC are key influencing factors that explain the variation in the C:N:P stoichiometry of leaves, fine roots, and litter. Compared with the C, N, and P contents in the aboveground parts of the forest, the soil properties had greater explanatory power on the first axis of the stoichiometric ratio. In this regional study, although soil is the direct supplier of nutrients required by plants, soil properties are the most important environmental factor. However, many other factors contribute to the observed variation, such as vegetation type, climate, and topography [84,85]. Adequate potassium (K) can enhance plant biomass production, increasing the quantity and quality of plant residues returned to the soil, thereby boosting soil C sequestration potential [86]. However, subsequent studies have revealed a negative correlation between soil K and soil C:N [87], and this contradiction may depend on AK levels. On the other hand, K helps regulate plant cellular water uptake, maintain osmotic balance, and promote the flow of water and nutrients between cells and tissues. Therefore, the stoichiometric relationships between K and C, N, and P may vary with water availability [88]. MBC, which represents the organic C content of the microbial community in the soil, reflects the activity of soil microbes and the organic matter decomposition process: soil microbes drive litter and organic matter decomposition, thus mediating nutrient release [32,89]. Under P limitation in limestone hill forests, microbes modulate enzyme production to optimize substrate utilization [16], with extracellular enzymes critically regulating soil organic matter nutrient dynamics [90,91]. Studies have shown that bacteria tend to utilize soil N and P, while fungi require larger amounts of C, and their residues are more resistant to decomposition [92]. Hence, microbial community structure may influence soil C and nutrient dynamics, leading to variations in aboveground C:N:P stoichiometric characteristics. Additionally, the symbiosis between arbuscular mycorrhizal fungi and plants can significantly enhance nutrient acquisition, such as by expanding the root absorption area through hyphae to promote nutrient uptake [93]. Thus, further studies should prioritize analyzing microbial C, N, and P acquisition enzymes and plant–fungal symbiotic relationships to advance the understanding of stoichiometric drivers.

## 4. Materials and Methods

### 4.1. Study Site

**Figure 6:**
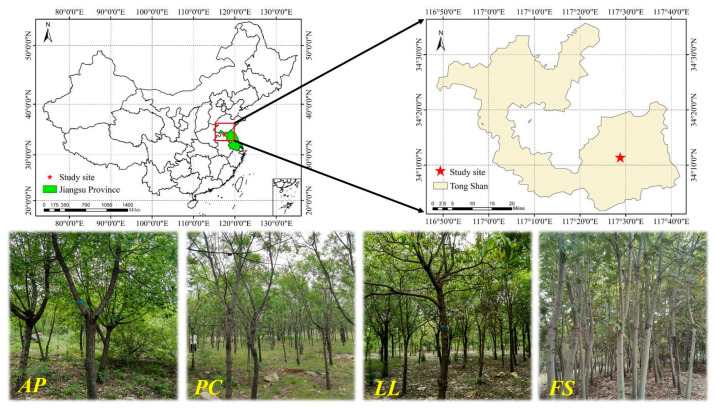
Location of the study area and field images of four typical forest stands.

The experimental site is located in Zhaotuan and Lvliang Forest Farms (34°01′–34°35′ N, 116°48′–117°42′ E) in Tongshan District, Xuzhou City, China (Figure 6). This area features hilly limestone terrain with rock coverage: 74% has a bare rock ratio of 0.3–0.8, 17% exceeds 0.8, and 9% is less than 0.3. The dominant soil type is Calcic Luvisol (IUSS Working Group WRB, 2022. Vienna, Austria), consisting predominantly of yellow-brown sandy loam with lower overall porosity (33–50%) and limited water retention capacity. It exhibits a relatively loose structure, featuring a top horizon of less than 15 cm. The climate is characterized by a temperate monsoon pattern with distinct seasons, a mean annual temperature of 14.5 °C, and an annual precipitation of 869 mm. Since the 1960s, afforestation activities have been carried out in the region under the support of Chinese greening projects, primarily consisting of artificial pure *PO* forests. Owing to differences in planting density and soil conditions, growth varies significantly. In areas with poor site quality and high planting density, tree vitality has declined progressively, accompanied by increasing mortality and dieback. The existing plant community is mainly composed of trees, including *PO*, *AP*, *PC*, *LL*, *FS*, *Melia azedarach*, etc. There are relatively few shrubs and herbaceous plants, with a distribution of *Broussonetia papyrifera*, *Celtis sinensis*, *Maclura tricuspidata*, *Rubus parvifolius*, *Clematis florida*, etc.

### 4.2. Experimental Design

The forest stands used in this study were artificially established on clear-cut sites of low-quality *PO* stands in approximately 2010. These include the pure forest of *AP*, *PC*, *LL*, and *FS*. *AP*, *PC*, and *FS* are deciduous trees, while *LL* is an evergreen tree (Figure 6). *AP*, *PC*, and *LL* exhibit strong stress resistance and have wide adaptability, whereas *FS* grows rapidly. They are primarily distributed in temperate regions. These plantations presented no understory shrubs or herbaceous vegetation, with only scattered seedlings of *PO* present. All the stands were established on soils of identical texture, with comparable stand ages (15–16 years). In June 2023, three 10 m × 10 m plots were established for each forest stand, totaling 12 plots. Basic information for each plot was obtained, including the diameter at breast height (DBH) and tree height (H) of all surviving trees, stand density, and canopy density (Table 2).

### 4.3. Data Collection

In July 2023, three standard trees were selected in each plot on the basis of the average DBH and average H. Using pole tree pruners, we collected healthy, undamaged, fully expanded fresh leaves from the mid-upper canopy layers in the four cardinal directions of each standard tree. Approximately 10 g of fine roots (diameter < 2 mm) were collected within 1 m of the trunk of each standard tree, following the methodology described by Berhongaray [94]. Along the plot diagonals, we established three 1 m × 1 m litter quadrats to collect all surface litter. For soil sampling, we employed the five-point sampling method: in each sampling plot, five collection points were established, one at the intersection of the plot’s diagonals (central point) and four additional points positioned equidistantly along each diagonal from the center. Soil samples were then collected using a soil auger from top to bottom at depths of 0–5 cm, 5–10 cm, and 10–20 cm. Soil samples from the same layer were homogenized to form composite samples for each of the three soil layers within the plot.

All collected samples were transported to the laboratory for processing. After removing soil impurities, the fine root samples were heat-inactivated with leaves at 105 °C for 2 h and then oven-dried with the litter samples at 75 °C to a constant weight. The C (g·kg^−1^) and N (g·kg^−1^) in plants and litter were measured using an elemental analyzer (PerkinElmer 2400 Series II CHNS/O Elemental Analyzer. PerkinElmer, Waltham, MA, USA), whereas P (g·kg^−1^) was determined through concentrated sulfuric acid–hydrogen peroxide digestion (applicable to plant and litter samples). Soil samples were passed through a 2 mm sieve to remove visible roots and other debris and then stored at room temperature to measure physical and chemical soil properties. Soil water content (SW, %) and microbial biomass were determined using fresh soil samples, while the remaining analyses were conducted with air-dried soil samples. We measured the SW by weighing the difference in the mass of the soil before and after it was dried in an oven and measured the soil pH with a digital pH meter. Available potassium (AK, mg·kg^−1^) in the soil was determined by atomic absorption spectrophotometry following CH_3_COONH_4_ solution extraction, whereas available phosphorus (AP, mg·kg^−1^) was determined by measuring the absorbance after extraction with the NaHCO_3_ solution and addition of the molybdenum–antimony–ascorbic acid chromogenic reagent. Microbial biomass reflects both soil nutrient status and its close association with forest C, N, and P cycling. The soil microbial biomass carbon (MBC, mg·kg^−1^) and nitrogen (MBN, mg·kg^−1^) contents were quantified using a total organic carbon analyzer after chloroform fumigation–extraction. Microbial biomass phosphorus (MBP, mg·kg^−1^) was extracted via chloroform fumigation followed by ultraviolet spectrophotometry. Some basic qualities of the soil across the different forest stands are shown in Table 3. The soil organic carbon (SOC, g·kg^−1^) content was quantified by potassium dichromate external heating, total soil N (TN, g·kg^−1^) was determined with an elemental analyzer, and total soil P (TP, g·kg^−1^) was measured using the concentrated sulfuric acid–perchloric acid digestion method (applicable to soil samples).

### 4.4. Statistical Analysis

One-way analysis of variance (ANOVA) was used to analyze the differences in the stoichiometric characteristics of the plants, litter, and soil between different forest stands, as well as the differences in the soil stoichiometry between the soil layers of the same forest stand. Significant differences were assessed via least significant difference (LSD) tests (*p* < 0.05), and the results are presented as means ± standard deviations (SDs). Two-way ANOVA was used to evaluate the effects of forest stand type, soil depth, and their interaction on soil stoichiometry. All analyses were conducted using IBM SPSS Statistics 26. Pearson correlation analysis in Origin 2024b was used to quantify the relationships between plantation component stoichiometry. Redundancy analysis (RDA) implemented in Canoco 5.0 was used to examine the influence of soil properties on aboveground stoichiometry, using Z-score standardized data with aboveground metrics as response variables and soil characteristics as explanatory variables. The results were visualized through biplot diagrams.

## 5. Conclusions

This study comprehensively analyzes the C, N, and P contents and stoichiometric relationships of the plant–litter–soil continuum in four artificial forests after the improvement of low-quality forests in limestone hills, as well as their response to soil factors. The results revealed that the C, N, and P contents and C:N:P ratios of the forests significantly differed. Compared with those of the other forest types, the leaves of *FS* presented higher N and P contents, whereas the litter of *LL* presented higher C:N and N:P ratios. The soil C, N, and P contents decreased with increasing soil depth, and both forest stand type and soil depth had highly significant effects on the soil C:N:P stoichiometry. Owing to its fast growth and high N and P requirements, *FS* adapts poorly to nutrient-poor soils. In contrast, other forest stands have lower leaf P contents than fine roots, with fine roots showing significant P enrichment, possibly employing a conservative survival strategy. Correlation analysis revealed that leaf N and P were negatively correlated with soil N, and fine root and litter P were also negatively correlated with 0–10 cm soil P. RDA revealed that soil AK, TN, and MBC were key drivers of stoichiometry in leaves, fine roots, and litter. The low soil P content in this region and the insufficient supply of P mean that the growth rate and productivity are limited primarily by P. Therefore, further forest stand improvements should prioritize the introduction of species adapted to low-P environments or those with strong P accumulation abilities, combined with P-based biological agents to activate insoluble P in soil to synergistically improve the ecological functions of limestone hills.

## Figures and Tables

**Table 1:** Influences of forest stand and soil depth on C, N, and P contents and stoichiometric ratios.

Factor	F (*p*) Value					
	C	N	P	C:N	N:P	C:P
Forest stand type	61.571 (<0.001)	108.526 (<0.001)	37.665 (<0.001)	0.937 (0.426)	10.447 (<0.001)	8.127 (<0.001)
Soil depth	223.808 (<0.001)	91.889 (<0.001)	57.648 (<0.001)	63.767 (<0.001)	17.790 (<0.001)	11.682 (<0.001)
Forest stand type × Soil depth	16.493 (<0.001)	7.919 (<0.001)	74.984 (<0.001)	4.965 (<0.001)	85.012 (<0.001)	84.003 (<0.001)

**Table 2:** Basic plot information.

Forest Stand	Age (a)	Canopy Density (%)	Average DBH (cm)	Average H (m)	Density (Tree·Ha)	Altitude (m)	Slope (°)
*AP*	16	0.69	8.9	4.8	2600	130	18
*PC*	15	0.54	8.7	5.3	2600	113	14
*LL*	15	0.60	9.2	8.0	3200	115	11
*FS*	16	0.82	8.1	4.2	2700	98	13

**Table 3:** Some basic qualities of the soil across the different forest stands.

Forest Stand	Soil Depth	pH	SW (%)	AP (mg·kg^−1^)	AK (mg·kg^−1^)	MBC (mg·kg^−1^)	MBN (mg·kg^−1^)	MBP (mg·kg^−1^)
*AP*	0–5 cm	7.26 ± 0.17 Aa	23.63 ± 1.91 Aa	2.77 ± 0.78 Aa	281.26 ± 4.77 Ba	451.99 ± 20.03 Cb	341.66 ± 16.07 Ba	10.55 ± 0.26 Ba
	5–10 cm	7.41 ± 0.14 Aa	23.16 ± 1.10 Aa	2.56 ± 0.49 ABa	268.59 ± 4.31 Ba	490.51 ± 45.81 Ca	352.28 ± 14.84 Ba	4.61 ± 0.30 Bb
	10–20 cm	7.34 ± 0.15 Aa	24.45 ± 1.18 Aa	1.98 ± 0.33 Bb	203.66 ± 9.19 Bb	314.52 ± 28.23 Cc	225.83 ± 24.40 Ab	10.9 ± 0.42 Aa
*PC*	0–5 cm	7.06 ± 0.22 Aa	25.72 ± 2.22 Aa	2.36 ± 0.28 Aa	214.06 ± 6.83 Ca	512.08 ± 58.65 Cab	309.45 ± 13.01 Ba	11.91 ± 0.99 Ba
	5–10 cm	7.23 ± 0.09 Aa	25.80 ± 1.80 Aa	2.21 ± 0.17 Ba	203.42 ± 6.08 Ca	532.31 ± 24.73 Ca	287.82 ± 8.28 Ca	5.22 ± 0.24 Bb
	10–20 cm	7.29 ± 0.11 Aa	26.83 ± 2.73 Aa	1.92 ± 0.12 Bb	186.16 ± 9.38 Ba	506.25 ± 20.59 Bb	198.08 ± 16.05 Bb	3.65 ± 0.64 Dc
*LL*	0–5 cm	7.26 ± 0.18 Aa	23.96 ± 2.11 Aa	3.48 ± 0.43 Aa	352.14 ± 37.29 Aa	1262.05 ± 141.57 Aa	326.25 ± 34.09 Ba	18.55 ± 3.05 Aa
	5–10 cm	7.24 ± 0.17 Aa	23.87 ± 1.95 Aa	3.31 ± 0.24 Aa	327.67 ± 18.49 Ab	1389.52 ± 15.51 Aa	337.61 ± 25.47 Ba	13.49 ± 1.18 Ab
	10–20 cm	7.23 ± 0.13 Aa	24.53 ± 2.19 Aa	2.63 ± 0.29 Ab	296.28 ± 9.84 Ac	1062.82 ± 57.86 Ab	111.22 ± 8.56 Cb	7.98 ± 0.52 Bc
*FS*	0–5 cm	7.20 ± 0.10 Aa	25.12 ± 3.31 Aa	2.82 ± 0.68 Aa	336.07 ± 14.12 Aa	955.51 ± 59.25 Bb	963.77 ± 41.28 Aa	19.76 ± 0.34 Aa
	5–10 cm	7.28 ± 0.11 Aa	24.81 ± 2.29 Aa	2.68 ± 0.67 ABa	311.28 ± 14.27 Ab	1144.49 ± 62.74 Ba	464.01 ± 39.09 Ab	3.09 ± 0.24 Cc
	10–20 cm	7.25 ± 0.13 Aa	25.96 ± 2.58 Aa	1.89 ± 0.26 Bb	277.93 ± 18.46 Ac	980.85 ± 51.88 Ab	237.16 ± 28.86 Ac	6.02 ± 0.51 Cb

Notes: Mean values ± standard error. Different uppercase letters indicate significant differences between forest stands within the same soil layer, whereas different lowercase letters indicate significant differences within the same forest stand between different soil layers (LSD, *p* < 0.05). SW, soil water; AP, available phosphorus; AK, available potassium; MBC, microbial biomass carbon; MBN, microbial biomass nitrogen; MBP, microbial biomass phosphorus.

## Data Availability

The raw data supporting the conclusions of this article will be made available by the authors on request.

## References

[1] Wei X., Liu R., Liu Y., He J., Chen J., Qi L., Zhou Y., Qin Y., Wu C., Dong J. (2024). Forest Areas in China Are Recovering Since the 21st Century. Geophys. Res. Lett..

[2] Cao S. (2011). Impact of China’s Large-Scale Ecological Restoration Program on the Environment and Society in Arid and Semiarid Areas of China: Achievements, Problems, Synthesis, and Applications. Crit. Rev. Environ. Sci. Technol..

[3] Shaaban M., Schwartz C., Macpherson J., Piorr A. (2021). A Conceptual Model Framework for Mapping, Analyzing and Managing Supply–Demand Mismatches of Ecosystem Services in Agricultural Landscapes. Land.

[4] Li L., Fan Z., Xiong K., Shen H., Guo Q., Dan W., Li R. (2021). Current situation and prospects of the studies of ecological industries and ecological products in eco-fragile areas. Environ. Res..

[5] Cao S., Chen L., Shankman D., Wang C., Wang X., Zhang H. (2011). Excessive reliance on afforestation in China’s arid and semi-arid regions: Lessons in ecological restoration. Earth-Sci. Rev..

[6] Elser J., Sterner R., Gorokhova E., Fagan W., Markow T., Cotner J., Harrison J., Hobbie S., Odell G., Weider L. (2000). Biological stoichiometry from genes to ecosystems. Ecol. Lett..

[7] Zechmeister-Boltenstern S., Keiblinger K.M., Mooshammer M., Peñuelas J., Richter A., Sardans J., Wanek W. (2015). The application of ecological stoichiometry to plant–microbial–soil organic matter transformations. Ecol. Monogr..

[8] Sinsabaugh R.L., Shah J.J.F. (2012). Ecoenzymatic Stoichiometry and Ecological Theory. Annu. Rev. Ecol. Evol. Syst..

[9] Zhou Z., Wang C., Luo Y. (2020). Meta-analysis of the impacts of global change factors on soil microbial diversity and functionality. Nat. Commun..

[10] Wan B., Barnes A.D., Potapov A., Yang J., Zhu M., Chen X., Hu F., Liu M. (2024). Altered litter stoichiometry drives energy dynamics of food webs through changing multiple facets of soil biodiversity. Soil Biol. Biochem..

[11] Ji W., LaZerte S.E., Waterway M.J., Lechowicz M.J. (2020). Functional ecology of congeneric variation in the leaf economics spectrum. New Phytol..

[12] Yan Z., Tian D., Han W., Tang Z., Fang J. (2017). An assessment on the uncertainty of the nitrogen to phosphorus ratio as a threshold for nutrient limitation in plants. Ann. Bot..

[13] Niklas K.J., Cobb E.D. (2005). N, P, and C stoichiometry of *Eranthis hyemalis* (Ranunculaceae) and the allometry of plant growth. Am. J. Bot..

[14] Yu Q., Wu H., He N., Lü X., Wang Z., Elser J.J., Wu J., Han X. (2012). Testing the Growth Rate Hypothesis in Vascular Plants with Above- and Below-Ground Biomass. PLoS ONE.

[15] Güsewell S. (2004). N:P ratios in terrestrial plants: Variation and functional significance. New Phytol..

[16] Cui Y., Moorhead D.L., Guo X., Peng S., Wang Y., Zhang X., Fang L. (2021). Stoichiometric models of microbial metabolic limitation in soil systems. Glob. Ecol. Biogeogr..

[17] Zeng Q., Lal R., Chen Y., An S. (2017). Soil, Leaf and Root Ecological Stoichiometry of Caragana korshinskii on the Loess Plateau of China in Relation to Plantation Age. PLoS ONE.

[18] Tu L.-H., Hu H.-L., Chen G., Peng Y., Xiao Y.-L., Hu T.-X., Zhang J., Li X.-W., Liu L., Tang Y. (2014). Nitrogen Addition Significantly Affects Forest Litter Decomposition under High Levels of Ambient Nitrogen Deposition. PLoS ONE.

[19] Bell C., Carrillo Y., Boot C.M., Rocca J.D., Pendall E., Wallenstein M.D. (2014). Rhizosphere stoichiometry: Are C:N:P ratios of plants, soils, and enzymes conserved at the plant species-level?. New Phytol..

[20] Xing S., Cheng X., Kang F., Wang J., Yan J., Han H. (2022). The patterns of N/P/K stoichiometry in the Quercus wutaishanica community among different life forms and organs and their responses to environmental factors in northern China. Ecol. Indic..

[21] Fan J., Harris W., Zhong H. (2016). Stoichiometry of leaf nitrogen and phosphorus of grasslands of the Inner Mongolian and Qinghai-Tibet Plateaus in relation to climatic variables and vegetation organization levels. Ecol. Res..

[22] Cui Y., Fang L., Guo X., Wang X., Zhang Y., Li P., Zhang X. (2018). Ecoenzymatic stoichiometry and microbial nutrient limitation in rhizosphere soil in the arid area of the northern Loess Plateau, China. Soil Biol. Biochem..

[23] Tipping E., Somerville C.J., Luster J. (2016). The C:N:P:S stoichiometry of soil organic matter. Biogeochemistry.

[24] Ott D., Digel C., Klarner B., Maraun M., Pollierer M., Rall B.C., Scheu S., Seelig G., Brose U. (2014). Litter elemental stoichiometry and biomass densities of forest soil invertebrates. Oikos.

[25] Yang Y., Liu B.-R., An S.-S. (2018). Ecological stoichiometry in leaves, roots, litters and soil among different plant communities in a desertified region of Northern China. CATENA.

[26] Li Y., Dong X., Yao W., Han C., Sun S., Zhao C. (2022). C, N, P, K stoichiometric characteristics of the “leaf-root-litter-soil” system in dryland plantations. Ecol. Indic..

[27] Blanco J.A., Durán M., Luquin J., Emeterio L.S., Yeste A., Canals R.M. (2023). Soil C/N ratios cause opposing effects in forests compared to grasslands on decomposition rates and stabilization factors in southern European ecosystems. Sci. Total. Environ..

[28] Kaźmierczak M., Błońska E., Lasota J. (2024). Effect of litter decomposition and nutrient release from shrub litter on enzymatic activity and C/N/P stoichiometry of soils in a temperate pine forest. Acta Oecologica.

[29] Zhao F., Kang D., Han X., Yang G., Yang G., Feng Y., Ren G. (2015). Soil stoichiometry and carbon storage in long-term afforestation soil affected by understory vegetation diversity. Ecol. Eng..

[30] Fan H., Wu J., Liu W., Yuan Y., Hu L., Cai Q. (2015). Linkages of plant and soil C:N:P stoichiometry and their relationships to forest growth in subtropical plantations. Plant Soil.

[31] Chen B., Chen L., Jiang L., Zhu J., Chen J., Huang Q., Liu J., Xu D., He Z. (2022). C:N:P Stoichiometry of Plant, Litter and Soil along an Elevational Gradient in Subtropical Forests of China. Forests.

[32] Wang L., Wang P., Sheng M., Tian J. (2018). Ecological stoichiometry and environmental influencing factors of soil nutrients in the karst rocky desertification ecosystem, southwest China. Glob. Ecol. Conserv..

[33] Wang W., Peng Y., Chen Y., Lei S., Wang X., Farooq T.H., Liang X., Zhang C., Yan W., Chen X. (2023). Ecological Stoichiometry and Stock Distribution of C, N, and P in Three Forest Types in a Karst Region of China. Plants.

[34] Zhang C., Zeng F., Zeng Z., Du H., Zhang L., Su L., Lu M., Zhang H. (2022). Carbon, Nitrogen and Phosphorus Stoichiometry and Its Influencing Factors in Karst Primary Forest. Forests.

[35] Tang Z., Xu W., Zhou G., Bai Y., Li J., Tang X., Chen D., Liu Q., Ma W., Xiong G. (2018). Patterns of plant carbon, nitrogen, and phosphorus concentration in relation to productivity in China’s terrestrial ecosystems. Proc. Natl. Acad. Sci. USA.

[36] Zhao H., Xu L., Wang Q., Tian J., Tang X., Tang Z., Xie Z., He N., Yu G. (2018). Spatial patterns and environmental factors influencing leaf carbon content in the forests and shrublands of China. J. Geogr. Sci..

[37] Tang J., Yin J., Qi J., Jepsen M., Lü X. (2012). Ecosystem carbon storage of tropical forests over limestone in Xishuang-banna, SW China. J. Trop. For. Sci..

[38] Han W.X., Fang J.Y., Guo D.L., Zhang Y. (2005). Leaf nitrogen and phosphorus stoichiometry across 753 terrestrial plant species in China. New Phytol..

[39] Reich P.B., Oleksyn J. (2004). Global patterns of plant leaf N and P in relation to temperature and latitude. Proc. Natl. Acad. Sci. USA.

[40] Chang Y., Zhong Q., Yang H., Xu C., Hua W., Li B. (2022). Patterns and driving factors of leaf C, N, and P stoichiometry in two forest types with different stand ages in a mid-subtropical zone. For. Ecosyst..

[41] Gong Z., Sheng M., Zheng X., Zhang Y., Wang L. (2023). Ecological stoichiometry of C, N, P and Si of Karst Masson pine forests: Insights for the forest management in southern China. Sci. Total. Environ..

[42] Hu Q., Sheng M., Bai Y., Jie Y., Xiao H. (2022). Response of C, N, and P stoichiometry characteristics of Broussonetia papyrifera to altitude gradients and soil nutrients in the karst rocky ecosystem, SW China. Plant Soil.

[43] Medina E., Cuevas E., Lugo A.E. (2017). Substrate Chemistry and Rainfall Regime Regulate Elemental Composition of Tree Leaves in Karst Forests. Forests.

[44] Hedin L.O. (2004). Global organization of terrestrial plant–nutrient interactions. Proc. Natl. Acad. Sci. USA.

[45] Liu L., Hu J., Chen X., Xu X., Yang Y., Ni J. (2022). Adaptation strategy of karst forests: Evidence from the community-weighted mean of plant functional traits. Ecol. Evol..

[46] Zhi X., Song Y., Yu D., Qian W., He M., Lin X., Zhang D., Gao S. (2023). Early Growth Characterization and C:N:P Stoichiometry in *Firmiana simplex* Seedlings in Response to Shade and Soil Types. Forests.

[47] Dibar D.T., Zhang K., Yuan S., Zhang J., Zhou Z., Ye X. (2020). Ecological stoichiometric characteristics of Carbon (C), Nitrogen (N) and Phosphorus (P) in leaf, root, stem, and soil in four wetland plants communities in Shengjin Lake, China. PLoS ONE.

[48] Yuan Z., Chen H.Y., Reich P.B. (2011). Global-scale latitudinal patterns of plant fine-root nitrogen and phosphorus. Nat. Commun..

[49] Ågren G.I. (2008). Stoichiometry and Nutrition of Plant Growth in Natural Communities. Annu. Rev. Ecol. Evol. Syst..

[50] Li L., Gao X., Gui D., Liu B., Zhang B., Li X. (2017). Stoichiometry in aboveground and fine roots of Seriphidium korovinii in desert grassland in response to artificial nitrogen addition. J. Plant Res..

[51] Guo Y., Yan Z., Gheyret G., Zhou G., Xie Z., Tang Z. (2020). The community-level scaling relationship between leaf nitrogen and phosphorus changes with plant growth, climate and nutrient limitation. J. Ecol..

[52] Nadelhoffer K.J. (2000). The potential effects of nitrogen deposition on fine-root production in forest ecosystems. New Phytol..

[53] Sardans J., Rivas-Ubach A., Peñuelas J. (2012). The C:N:P stoichiometry of organisms and ecosystems in a changing world: A review and perspectives. Perspect. Plant Ecol. Evol. Syst..

[54] Bradshaw C., Kautsky U., Kumblad L. (2012). Ecological Stoichiometry and Multi-element Transfer in a Coastal Ecosystem. Ecosystems.

[55] Wang Z., Lu J., Yang M., Yang H., Zhang Q. (2015). Stoichiometric Characteristics of Carbon, Nitrogen, and Phosphorus in Leaves of Differently Aged Lucerne (*Medicago sativa*) Stands. Front. Plant Sci..

[56] Wang Z., Lv S., Song H., Wang M., Zhao Q., Huang H., Niklas K.J. (2020). Plant type dominates fine-root C:N:P stoichiometry across China: A meta-analysis. J. Biogeogr..

[57] Drenovsky R., Richards J. (2004). Critical N:P values: Predicting nutrient deficiencies in desert shrublands. Plant Soil.

[58] Paudel E., Dossa G.G., Xu J., Harrison R.D. (2015). Litterfall and nutrient return along a disturbance gradient in a tropical montane forest. For. Ecol. Manag..

[59] Bowden R.D., Wurzbacher S.J., Washko S.E., Wind L., Rice A.M., Coble A.E., Baldauf N., Johnson B., Wang J., Simpson M. (2019). Long-term Nitrogen Addition Decreases Organic Matter Decomposition and Increases Forest Soil Carbon. Soil Sci. Soc. Am. J..

[60] Huang X., Lu Z., Xu X., Wan F., Liao J., Wang J. (2023). Global distributions of foliar nitrogen and phosphorus resorption in forest ecosystems. Sci. Total. Environ..

[61] Killingbeck K.T. (1996). Nutrients in Senesced Leaves: Keys to the Search for Potential Resorption and Resorption Proficiency. Ecology.

[62] Liu R., Wang D. (2021). C:N:P stoichiometric characteristics and seasonal dynamics of leaf-root-litter-soil in plantations on the loess plateau. Ecol. Indic..

[63] Cotrufo M.F., Wallenstein M.D., Boot C.M., Denef K., Paul E. (2013). The Microbial Efficiency-Matrix Stabilization (MEMS) framework integrates plant litter decomposition with soil organic matter stabilization: Do labile plant inputs form stable soil organic matter?. Glob. Change Biol..

[64] Aerts R. (1996). Nutrient Resorption from Senescing Leaves of Perennials: Are there General Patterns?. J. Ecol..

[65] Liang S., Tan T., Wu D., Li C., Jing H., Wu J. (2023). Seasonal variations in carbon, nitrogen, and phosphorus of Pinus yunnanenis at different stand ages. Front. Plant Sci..

[66] Tian H., Chen G., Zhang C., Melillo J.M., Hall C.A.S. (2010). Pattern and variation of C:N:P ratios in China’s soils: A synthesis of observational data. Biogeochemistry.

[67] Qiao Y., Wang J., Liu H., Huang K., Yang Q., Lu R., Yan L., Wang X., Xia J. (2020). Depth-dependent soil C-N-P stoichiometry in a mature subtropical broadleaf forest. Geoderma.

[68] Yu Z., Wang M., Huang Z., Lin T., Vadeboncoeur M.A., Searle E.B., Chen H.Y.H. (2018). Temporal changes in soil C-N-P stoichiometry over the past 60 years across subtropical China. Glob. Change Biol..

[69] Eaton J.M., McGoff N.M., Byrne K.A., Leahy P., Kiely G. (2008). Land cover change and soil organic carbon stocks in the Republic of Ireland 1851–2000. Clim. Change.

[70] Fu X., Shao M., Wei X., Horton R. (2010). Soil organic carbon and total nitrogen as affected by vegetation types in Northern Loess Plateau of China. Geoderma.

[71] Brindhavani P.M., Chitdeshwari T., Selvi D., Sivakumar U., Jeyakumar P. (2022). Solubilization of phosphorus by low molecular weight organic acids and amino acids in calcareous soils: LMWOA and amino acid on P solubilization. J. Appl. Nat. Sci..

[72] Taalab A.S., Ageeb G.W., Siam H.S., Mahmoud S.A. (2019). Some characteristics of calcareous soils. A review. Middle East J. Agric. Res..

[73] Siswanto D., Widjajani B.W., Siswanto S. (2024). Analisis Status dan Kelas Kemampuan Kesuburan Tanah pada Beberapa Lahan Tebu di Kecamatan Japah Kabupaten Blora. J. Agrotropika.

[74] Hobbie S.E. (2015). Plant species effects on nutrient cycling: Revisiting litter feedbacks. Trends Ecol. Evol..

[75] Liu Y., Fang Y., An S. (2020). How C:N:P stoichiometry in soils and plants responds to succession in Robinia pseudoacacia forests on the Loess Plateau, China. For. Ecol. Manag..

[76] Yang Y., Liu B. (2019). Effects of planting Caragana shrubs on soil nutrients and stoichiometries in desert steppe of Northwest China. CATENA.

[77] Błońska E., Lasota J., Prażuch W., Ilek A. (2024). Vertical variations in enzymatic activity and C:N:P stoichiometry in forest soils under the influence of different tree species. Eur. J. For. Res..

[78] Zhao F., Sun J., Ren C., Deng J., Han X., Yang G., Feng Y., Ren G. (2015). Land use change influences soil C, N and P stoichiometry under ‘Grain-to-Green Program’ in China. Sci. Rep..

[79] Ladanai S., Ågren G.I., Olsson B.A. (2010). Relationships Between Tree and Soil Properties in Picea abies and Pinus sylvestris Forests in Sweden. Ecosystems.

[80] Bui E.N., Henderson B.L. (2013). C:N:P stoichiometry in Australian soils with respect to vegetation and environmental factors. Plant Soil.

[81] Zeng Q., Li X., Dong Y., An S., Darboux F. (2016). Soil and plant components ecological stoichiometry in four steppe communities in the Loess Plateau of China. CATENA.

[82] He M., Dijkstra F.A., Zhang K., Tan H., Zhao Y., Li X. (2016). Influence of life form, taxonomy, climate, and soil properties on shoot and root concentrations of 11 elements in herbaceous plants in a temperate desert. Plant Soil.

[83] Robbins C.J., Matthaeus W.J., Cook S.C., Housley L.M., Robison S.E., Garbarino M.A., LeBrun E.S., Raut S., Tseng C., King R.S. (2019). Leaf litter identity alters the timing of lotic nutrient dynamics. Freshw. Biol..

[84] Ordoñez J.C., Van Bodegom P.M., Witte J.M., Wright I.J., Reich P.B., Aerts R. (2009). A global study of relationships between leaf traits, climate and soil measures of nutrient fertility. Glob. Ecol. Biogeogr..

[85] Zhang J., Zhao N., Liu C., Yang H., Li M., Yu G., Wilcox K., Yu Q., He N. (2018). C:N:P stoichiometry in China’s forests: From organs to ecosystems. Funct. Ecol..

[86] Tripler C.E., Kaushal S.S., Likens G.E., Todd Walter M. (2006). Patterns in potassium dynamics in forest ecosystems. Ecol. Lett..

[87] Chen Z., Jiang Z., Li Q., Tan Y., Yan P., Arif M. (2024). Examining the stoichiometry of C:N:P:K in the dynamics of foliar-litter-soil within dominant tree species across different altitudes in southern China. Glob. Ecol. Conserv..

[88] Sardans J., Peñuelas J., Coll M., Vayreda J., Rivas-Ubach A. (2012). Stoichiometry of potassium is largely determined by water availability and growth in Catalonian forests. Funct. Ecol..

[89] Burns R.G., DeForest J.L., Marxsen J., Sinsabaugh R.L., Stromberger M.E., Wallenstein M.D., Weintraub M.N., Zoppini A. (2013). Soil enzymes in a changing environment: Current knowledge and future directions. Soil Biol. Biochem..

[90] Sinsabaugh R.L., Lauber C.L., Weintraub M.N., Ahmed B., Allison S.D., Crenshaw C., Contosta A.R., Cusack D., Frey S., Gallo M.E. (2008). Stoichiometry of soil enzyme activity at global scale. Ecol. Lett..

[91] Allison S.D., Czimczik C.I., Treseder K.K. (2008). Microbial activity and soil respiration under nitrogen addition in Alaskan boreal forest. Glob. Change Biol..

[92] Keiblinger K.M., Hall E.K., Wanek W., Szukics U., Hämmerle I., Ellersdorfer G., Böck S., Strauss J., Sterflinger K., Richter A. (2010). The effect of resource quantity and resource stoichiometry on microbial carbon-use-efficiency. FEMS Microbiol. Ecol..

[93] He S., Long M., He X., Guo L., Yang J., Yang P., Hu T. (2017). Arbuscular mycorrhizal fungi and water availability affect biomass and C:N:P ecological stoichiometry in alfalfa (*Medicago sativa* L.) during regrowth. Acta Physiol. Plant..

[94] Berhongaray G., Janssens I.A., King J.S., Ceulemans R. (2013). Fine root biomass and turnover of two fast-growing poplar genotypes in a short-rotation coppice culture. Plant Soil.

